# Bayesian Linear Inverse Problems in Regularity Scales with Discrete Observations

**DOI:** 10.1007/s13171-024-00342-0

**Published:** 2024-03-07

**Authors:** Dong Yan, Shota Gugushvili, Aad van der Vaart

**Affiliations:** 1 DIAM, TU Delft, Mekelweg 4, Delft, 2628 CD Netherlands; 2 Biometris, Plant Sciences Group, Wageningen University & Research, P.O. Box 16, Wageningen, 6700 AA The Netherlands

**Keywords:** Adaptive estimation, Gaussian prior, Hilbert scale, Linear inverse problem, Nonparametric Bayesian estimation, Posterior contraction rate, Random series prior, Regularity scale, Regression, Fixed design, Interpolation, Galerkin, 62G20, 35R30

## Abstract

We obtain rates of contraction of posterior distributions in inverse problems with discrete observations. In a general setting of smoothness scales we derive abstract results for general priors, with contraction rates determined by discrete Galerkin approximation. The rate depends on the amount of prior concentration near the true function and the prior mass of functions with inferior Galerkin approximation. We apply the general result to non-conjugate series priors, showing that these priors give near optimal and adaptive recovery in some generality, Gaussian priors, and mixtures of Gaussian priors, where the latter are also shown to be near optimal and adaptive.

## Introduction

In an inverse problem one observes a noisy version of a transformed signal *Af* and wishes to recover the unknown signal *f*. The *forward operator* *A* is assumed to be injective, but its inverse is discontinuous, so that a naive inversion would amplify the noise. In this paper we study a Bayesian method for regularising the inverse in a setting with discrete observations. We assume that the transformed signal is a function $Af: \mathcal {D} \rightarrow \mathbb {R}$ on a bounded domain $\mathcal {D} \subset \mathbb {R}^d$, and for given *design points* $\{x_1,\ldots ,x_n \} \subset \mathcal {D}$, we observe $Y^n=(Y_1,\ldots , Y_n)$ defined by

1 $$\begin{aligned} Y_i = Af(x_i) + Z_i, \qquad i = 1,\cdots , n, \end{aligned}$$

with $Z_i$ i.i.d. standard normal random variables. This *regression model* (1) may be compared to its continuous version, the *white noise model*, in which the observation is given by

2 $$\begin{aligned} Y^{(n)} = A f + \frac{1}{\sqrt{n}} \xi , \end{aligned}$$

where $\xi$ is Gaussian (white) noise. There is a rich literature on the continuous problem, even though the regression problem is more realistic in practice.

The review papers Cavalier (2008); Stuart (2010) provide an overview to the field of inverse problems up to the time around 2010. The former paper focuses on frequentist studies, and the latter one concentrates on Bayesian methods, as does the present paper. The book Nickl (2023) gives a recent overview, including nonlinear problems. Bayesian methods for the white noise problem (2) were studied in among others Knapik et al. (2011, 2013); Knapik and Salomond (2018); Gugushvili et al. (2020); Agapiou et al. (2013), but the regression model is less explored, except in the case that *A* is identity (e.g. Sniekers and van der Vaart (2015, 2020)). In Gugushvili et al. (2018) Gaussian conjugacy was used to study a problem with one-dimensional $\mathcal D$.

The Bayesian approach consists of putting a *prior* probability measure on *f*, and, after collecting the data, updating this to the *posterior* probability measure. If $y\mapsto p_f^{(n)}(y)$ denotes the (Gaussian) density of the data $Y^n=(Y_1,\ldots , Y_n)$, then this is the Borel measure on *H*, given, by Bayes’s formula, as

3 $$\begin{aligned} {\mathrm {\Pi }}_n(f \in B \,|\ Y^n) = \frac{\int _B p_f^{(n)} (Y^n) \,d{\mathrm {\Pi }}_n(f) }{\int p_f^{(n)} (Y^n)\, d{\mathrm {\Pi }}_n(f)}. \end{aligned}$$

In the Bayesian paradigm the posterior distribution encompasses all the necessary information for inference on *f*. An attractive feature of the Bayesian approach is that it not only offers an estimation procedure, through a measure of ‘centre’ of the posterior distribution, but also provides a way to conduct uncertainty quantification, through the spread of the posterior distribution.

If one thinks of the Bayesian approach as a method and not merely as a philosophy, then one wants the posterior measure (3) to recover the function $f_0$ if in reality $Y_1,\ldots , Y_n$ are generated according to the model (1) with $f=f_0$ and $n\rightarrow \infty$. We shall be interested in the *rate of posterior contraction*. Following Ghosal et al. (2000); Ghosal and van der Vaart (2007, 2017), we say that a sequence $\varepsilon _{n}\downarrow 0$ is a rate of posterior contraction to $f_0$ if, for a fixed sufficiently large constant *M*, and $n\rightarrow \infty$,

4 $$\begin{aligned} {\mathrm {\Pi }}_n\bigl (f: \Vert f-f_0\Vert _{}>M \varepsilon _{n} \,|\ Y^n\bigr ) \overset{\mathbb {P}^{(n)}_{f_0} }{\rightarrow } 0. \end{aligned}$$

We are interested in the fastest rate for which this is satisfied, and compare this to the rate of estimation of non-Bayesian methods.

The paper is organised as follows. Section 2 introduces our setup along with the assumptions, and introduces an interpolation device. Section 3 presents two general theorems, which are applied to two special cases, series priors and (mixtures of) Gaussian priors, in Sections 4 and 5. Section 6 contains some of the proofs.

The paper extends results for the white noise model obtained in Gugushvili et al. (2020) to the case of discrete observations. While we repeat necessary definitions, we refer to the latter paper for further examples and discussion.

### Notation

The symbols $\lesssim , \gtrsim , \simeq$ mean $\le , \ge , =$ up to a positive multiple independent of *n*, (or another asymptotic parameter). The constant may be stated explicitly in subscripts, and e.g. $\lesssim _f$ means that it depends on *f*.

## Setup

In this section we formulate the assumptions on the operator *A* and present the interpolation device to handle the discrete observational scheme. The first part of the section borrows from Gugushvili et al. (2020).

### Smoothing

The function *f* in (1) is an element of a Hilbert space $(H, \langle \cdot ,\cdot \rangle )$. We embed this space as the space $H=H_0$ in a ‘scale of smoothness classes’, as is common in the literature on inverse problems, as follows.

#### Definition 2.1

(Smoothness scale). For every $s\in \mathbb {R}$ the space $H_s$ is an infinite-dimensional, separable Hilbert space, with inner product $\langle \cdot ,\cdot \rangle _{s}$ and induced norm $\Vert \cdot \Vert _{s}$. The spaces $(H_s)_{s\in \mathbb {R}}$ satisfy the following conditions:

1. For $s<t$ the space $H_t$ is a dense subspace of $H_s$ and $\Vert f\Vert _s \lesssim \Vert f\Vert _t$, for $f\in H_t$.
2. For $s\ge 0$ and $f\in H_0$ viewed as element of $H_{-s}\supset H_0$,
  5 $$\begin{aligned} \Vert f\Vert _{-s}=\sup _{\Vert g\Vert _s\le 1}\langle f,g\rangle _{}, \qquad f\in H_0. \end{aligned}$$

#### Assumption 2.2

(Approximation). For every $j\in \mathbb {N}$ and $s\in (0,S)$, for some $S>0$, there exists a $(j-1)$-dimensional linear subspace $V_j\subset H_0$ and a number $\delta (j,s)$ such that $\delta (j,s) \rightarrow 0$ as $j\rightarrow \infty$, and such that

6 $$\begin{aligned} \inf _{g \in V_j} \Vert f - g\Vert _{}{} & {} \lesssim \delta (j,s)\, \Vert f\Vert _s,\end{aligned}$$

7 $$\begin{aligned} \Vert g\Vert _s{} & {} \lesssim \frac{1}{\delta (j,s)}\,\Vert g\Vert _{},\qquad \forall g\in V_j. \end{aligned}$$

The two inequalities (6) and (7) are known as inequalities of Jackson and Bernstein type, respectively, see, e.g., Canuto et al. (2010). The approximation property (6) shows that ‘smooth elements’ $f\in H_s$ are well approximated in $\Vert \cdot \Vert _{}$ by their projection onto a finite-dimensional space $V_j$, with approximation error tending to zero as the dimension of $V_j$ tends to infinity. We shall focus on the case of polynomial dependence $\delta (j,s)\simeq j^{-s/d}$, where *d* is the dimension of the domain of the functions in $H_0$.

The Sobolev spaces $H_s(\mathcal {D})=W^{s,2}(\mathcal {D})$ provide perhaps the most important example of a smoothness scale (see e.g. Triebel (2010, 2008); Haroske and Triebel (2008)), which can also be restricted by boundary conditions, as common in the theory of differential equations. The following example gives a smoothness scale described through basis functions. Because one can always choose an appropriate basis, for our purposes not much is lost by considering only this concrete example.

#### Example 2.3

(Sequence spaces). Suppose $(\phi _i)_{i\in \mathbb {N}}$ is a given orthonormal sequence in the Hilbert *H*, and $1\le b_{i} \uparrow \infty$ is a given sequence of numbers. For $s\ge 0$, define $H_s$ as the set of all elements $f=\sum _{i\in \mathbb {N}} f_i \phi _i\in H$ with $\sum _{i\in \mathbb {N}}b_i^{2s}f_i^2<\infty$, equipped with the norm

$$\begin{aligned} \Vert f\Vert _s=\Bigl (\sum \limits _{i\in \mathbb {N}}b_i^{2s}f_i^2\Bigr )^{1/2}. \end{aligned}$$

For $s<0$, equip the elements $f=\sum _{i=1}^\infty f_i\phi _i$ of *H*, where $(f_i)\in \ell _2$, with the norm as in the display, which is now automatically finite, and next define $H_s$ as the completion of *H* under this norm.

It can be checked that these spaces form a smoothness scale as in Definition 2.1 and that Assumption 2.2 is satisfied with the spaces $V_j=\textsf{Span}(\phi _i: i<j)$, and the approximation numbers $\delta (j,s)= b_j^{-s}$.

The forward operator *A* in the model (1) is assumed to be a bounded linear operator $A: H\rightarrow G$ between the separable Hilbert spaces *H* and *G*, which is ‘smoothing’. The following assumption, which is standard in the literature (see Cohen et al. (2004); Natterer (1984); Goldenshluger and Pereverzev (2003); Mathé and Pereverzev (2001)) makes this precise.

#### Assumption 2.4

(Smoothing property of *A*). For some $\gamma >0$, the operator $A: H_{-\gamma } \rightarrow G$ is injective and bounded and, for every $f\in H_0$,

8 $$\begin{aligned} \Vert A f\Vert _{}\simeq \Vert f\Vert _{-\gamma }. \end{aligned}$$

While the preceding assumption is sufficient for our main results, a stronger smoothing property is often satisfied and may be helpful when analysing particular priors. We now assume that both $H=H_0$ and $G=G_0$ are embedded are embedded in smoothness scales $(H_s)_{s\in \mathbb {R}}$ and $(G_s)_{s\in \mathbb {R}}$ as in Definition 2.1. Somewhat abusing notation, we write the norms of both scales as $\Vert \cdot \Vert _s$.

#### Assumption 2.5

(Strengthened smoothing property of *A*). For some $\gamma >0$, the operator $A: H_{s-\gamma } \rightarrow G_s$ is injective and bounded for every $s\ge 0$, and, for every $f\in H_0 \cap H_{s-\gamma }$,

9 $$\begin{aligned} \Vert A f\Vert _s \simeq \Vert f\Vert _{s-\gamma }. \end{aligned}$$

#### Example 2.6

(SVD). If the operator $A: H\rightarrow G$ is compact, then the positive self-adjoint operator $A^*A: H\rightarrow H$ possesses a countable orthonormal basis of eigenfunctions $\phi _i$, which can be arranged so that the corresponding sequence of eigenvalues $\lambda _{i}$ decreases to zero. If *A* is injective, then all eigenvalues, whose roots are known as the *singular values* of *A*, are strictly positive. Suppose that there exists $\gamma >0$ such that they are of order

$$\begin{aligned} \lambda _{i}\simeq i^{-2\gamma }. \end{aligned}$$

If we construct the smoothness classes $(H_s)_{s\in \mathbb {R}}$ from the basis $(\phi _i)_{i\in \mathbb {N}}$ and the numbers $b_i=i$ as in Example 2.3, then (8) is satisfied, since $\Vert Af\Vert ^2=\langle A^TAf,f\rangle =\sum _{i} \lambda _{i}^2f_i^2\simeq \Vert f\Vert _{-\gamma }^2$.

If we also construct smoothness classes $(G_s)_{s\in \mathbb {R}}$ from the orthonormal sequence $U\phi _i$ in *G*, where $U: \textrm{Range}(A)\rightarrow G$ is the partial isometry in the polar decomposition $Af= U(A^*A)^{1/2}f$ of *A*, then (9) will also be satisfied, for every $s\ge 0$.

Concrete examples of operators *A* satisfying Assumptions 2.4 or 2.5 can be found in Gugushvili et al. (2020).

### Interpolation

The sampling scheme (1) collects discrete data, but the operator *A* acts on a continuous function, which we wish to estimate on its full domain. In this section we describe an interpolation technique that maps discrete signals to the continuous domain. A similar technique has been used in the context of proving asymptotic equivalence of the white noise model and nonparametric regression; see Reiß (2008) and the references therein. The assumptions are also inspired by the theory from the field of numerical analysis, see Canuto et al. (2010).

The range space *G* of the operator $A: H\rightarrow G$ is a collection of functions $g: \mathcal {D} \rightarrow \mathbb {R}$, equipped with a pre-inner product $\langle \cdot , \cdot \rangle$. The design points give rise to a “discrete” semi-inner product and semi-norm on *G* given by

$$\begin{aligned} \langle g, h \rangle _{n} := \frac{1}{n} \sum \limits _{i=1}^{n} g(x_i)h(x_i) \qquad \Vert g\Vert _{n} := \sqrt{\frac{1}{n} \sum \limits _{i=1}^{n} g^2(x_i)}. \end{aligned}$$

(The notation $\Vert \cdot \Vert _n$ clashes with the notation $\Vert \cdot \Vert _s$ for the norms of the smoothness scales, but this should not lead to confusion as *n* will never appear as smoothness level.)

For every $n\in \mathbb {N}$, we fix an *n*-dimensional subspace $\widetilde{W}_n\subset G$, and, for $g\in G$, denote by $\mathcal {I}_ng$ the element of $\widetilde{W}_n$ that interpolates *g* at the design points, i.e.

$$\begin{aligned} \mathcal {I}_ng(x_i)=g(x_i), \qquad i=1,\ldots , n. \end{aligned}$$

This defines a map $\mathcal {I}_n: G\rightarrow \widetilde{W}_n\subset G$. Existence and uniqueness of this map is addressed in Lemma 2.9 below. (In order that the point evaluations $g(x_i)$ be well defined, the domain *G* may be restricted to continuous functions.)

#### Assumption 2.7

(Interpolation). For every $n\in \mathbb {N}$ and $s\in (s_d,S_d)$, for some $S_d>s_d\ge 0$, there exist an *n*-dimensional subspace $\widetilde{W}_n\subset G$ and a number $\delta _{d}(n,s)$ such that

10 $$\begin{aligned} \Vert w\Vert _{n}{} & {} \simeq \Vert w\Vert ,\qquad \forall w\in \widetilde{W}_n,\end{aligned}$$

11 $$\begin{aligned} \Vert \mathcal {I}_n g - g \Vert{} & {} \lesssim \delta _{d}(n,s) \,\Vert g\Vert _s. \end{aligned}$$

In this assumption $\Vert \cdot \Vert _s$ are the norms of a smoothness scale $(G_s)_{s\in \mathbb {R}}$ as in Definition 2.1, in which the space *G* is embedded as $G=G_0$ The approximation numbers $\delta _{d}(n,s)$ will often be the same as the approximation rates $\delta (n,s)$ in Assumption 2.2. However, the approximation (11) is typically not true for every smoothness level $s>0$, but only for *s* in a range $(s_d,S_d)$. For instance, for Sobolev scales the lower bound $s_d$ is typically equal to *d*/2 for *d* the dimension of the domain $\mathcal {D}$ of the functions in *G*, and the upper bound $S_d$ is the regularity of the basis elements used to define the scale.

Fix an arbitrary orthonormal basis $e_{1,n},\ldots , e_{n,n}$ of $\widetilde{W}_n$ relative to the discrete inner product $\langle \cdot ,\cdot \rangle _n$, and given the discrete data $(Y_1,\ldots , Y_n)$ as in (1), define

12 $$\begin{aligned} Y^{(n)}=\sum \limits _{i=1}^{n} \frac{1}{n} \sum \limits _{j=1}^{n} Y_je_{i,n}(x_j) \,e_{i,n}. \end{aligned}$$

This embeds the discrete data as a ‘continuous signal’ into the space $\widetilde{W}_n\subset G$. If the observations satisfy (1), then the continuous observation $Y^{(n)}$ can be decomposed as

$$\begin{aligned} Y^{(n)}=\sum \limits _{i=1}^{n} \langle Af, e_{i,n}\rangle _ne_{i,n}+ \frac{1}{n} \sum \limits _{j=1}^{n} Z_{j} \sum \limits _{i=1}^{n} e_{i,n}(x_j) \,e_{i,n} =\mathcal {I}_n Af+\frac{1}{\sqrt{n}}\xi ^{(n)}, \end{aligned}$$

where $\xi ^{(n)}$ is a Gaussian random variable with values in the space in $\widetilde{W}_n$. Loosely speaking, as $n\rightarrow \infty$ the operators $\mathcal {I}_n$ should tend to the identity operator, and the mean of the signal $Y^{(n)}$ should become more representative of the full signal *Af*. As shown in the following lemma, the noise $\xi ^{(n)}$ tends to white noise in the Hilbert space *G*.

#### Lemma 2.8

The variable $\xi ^{(n)}$ defined in the preceding display is a Gaussian random element in $\widetilde{W}_n\subset G$ with mean zero. Under (10) its covariance operator is up to multiplicative constants that do not depend on *n* bounded below and above by the orthogonal projection $\tilde{Q}_n: G\rightarrow \widetilde{W}_n$ relative to the continuous inner product $\langle \cdot ,\cdot \rangle$.

#### Proof

For $g\in G$, we can write $\langle \xi ^{(n)},g\rangle \!=\! n^{-1/2} \sum _{j=1}^{n} Z_{j} \sum _{i=1}^{n} e_{i,n}(x_j)\langle e_{i,n},g\rangle$. The expectation of this variable vanishes, while the variance is given by

$$\begin{aligned} \textrm{var}\langle \xi ^{(n)},g\rangle \!=\! \frac{1}{n} \sum \limits _{j=1}^{n} \Bigl (\sum \limits _{i=1}^{n} e_{i,n}(x_j)\langle e_{i,n},g\rangle \Bigr )^2 \!= \Bigr \Vert \sum \limits _{i=1}^n \langle e_{i,n},g\rangle e_{i,n}\Bigl \Vert _n^2\!=\!\sum \limits _{i=1}^n \langle e_{i,n},g\rangle ^2. \end{aligned}$$

by the orthogonality of the basis.

The orthogonal projection $\tilde{Q}_ng$ of *g* onto $\widetilde{W}_n$ relative to the continuous inner product is equal to $\tilde{Q}_ng=\sum _{i=1}^{n}\alpha _{i} e_{i,n}$, for $\alpha =(\alpha _{1},\ldots , \alpha _{n})^T= \Sigma _n^{-1} \beta$, where $\beta =\bigl (\langle e_{i,n},g\rangle \bigr )_{i=1..n}$ and $\Sigma _n$ is the Gram matrix $\bigl (\langle e_{i,n},e_{j,n}\rangle \bigr )$. Hence $\Vert \tilde{Q}_ng\Vert ^2=\alpha ^{T}\Sigma _{n}\alpha = \beta ^T\Sigma _n^{-1}\beta$. Because $\Sigma _n$ is bounded above and below by a multiple of the identity, by Lemma 2.9 below, it follows that $\Vert \tilde{Q}_ng\Vert ^2\simeq \beta ^{T} \beta = \sum _{i=1}^n \langle e_{i,n},g\rangle ^2$.

#### Lemma 2.9

Suppose that $\widetilde{W}_n\subset G$ is a finite-dimensional subspace so that (10) holds. Then for every $g\in G$ there exists a function $\mathcal {I}_ng\in \widetilde{W}_n$ that interpolates *g* at the points $x_1,\ldots , x_n$ and

- $\mathcal {I}_ng$ is the orthogonal projection of *g* onto $\widetilde{W}_n\subset G$ relative to $\langle \cdot ,\cdot \rangle _n$.
- $$\Vert g\Vert _n\simeq \Vert \mathcal {I}_ng\Vert $$, for every $$g\in G$$.

Furthermore, for any orthonormal basis $e_{1,n},\ldots , e_{n,n}$ of $\widetilde{W}_n$ relative to $\langle \cdot ,\cdot \rangle _n$, the Gram matrix $\bigl (\langle e_{i,n}, e_{j,n}\rangle \bigr ){}_{i,j=1..n}$ is bounded above and below by the identity matrix times a constant that does not depend on *n*.

#### Proof

Under (10), the linear map $R: \widetilde{W}_n\subset G\rightarrow \mathbb {R}^{n}$ given by $(Rw)_i=w(x_i)$, for $i=1,\ldots ,n$, has kernel 0 and hence is a bijection. Thus there is a solution $w=\mathcal {I}_ng\in \widetilde{W}_n$ to $R w=\big (g(x_i)\bigr )_{i=1..n}$, for every $g\in G$, and this is unique.

The function $\mathcal {I}_ng$ is contained in $\widetilde{W}_n$ by definition, and satisfies $\langle g-\mathcal {I}_ng,h\rangle _n=n^{-1} \sum _{i=1}^{n} g(x_i)h(x_i)-n^{-1} \sum _{i=1}^{n} \mathcal {I}_ng(x_i)h(x_i)=0$, for every $h\in \widetilde{W}_n$. Thus $\mathcal {I}_ng$ is the orthogonal projection of *g* onto $\widetilde{W}_n$ relative to $\langle \cdot ,\cdot \rangle _n$. Because the functions *g* and $\mathcal {I}_ng$ coincide at the design points, we have $\Vert g\Vert _n=\Vert \mathcal {I}_ng\Vert _n$, which is equivalent to $\Vert \mathcal {I}_ng\Vert$, by (10).

The final statement is the equivalence $\Vert \sum _{i=1}^{n}\alpha _{i}e_{i,n}\Vert ^2\simeq \alpha ^{T}\alpha$, for $\alpha \in \mathbb {R}^{n}$. Now $\Vert \sum _{i=1}^{n}\alpha _{i}e_{i,n}\Vert \simeq \Vert \sum _{i=1}^{n}\alpha _{i}e_{i,n}\Vert _n$, by (10), and this is equal to the Euclidean norm of $\alpha$ if $e_{1,n},\ldots , e_{n,n}$ is orthonormal relative to $\langle \cdot ,\cdot \rangle _n$.

The following two examples exhibit suitable discretisation spaces, both with equidistant design points.

#### Example 2.10

(Trigonometric Polynomials). This example is adapted from Section 2.3 in Reiß (2008). Let $\mathcal {D} = \mathbb {T}^d = (0,1]^d$, for $d\in \mathbb {N}$, and consider the set of $n=m^d$ design points $\{k/m\}_{k\in \{1,\cdots ,m\}^d}$, for a given odd natural number *m*. In this case, the Fourier system

$$\begin{aligned} e_k (x) = e^{\textsf{i} 2\pi \langle k,x \rangle _{\mathbb {R}^d} }, \qquad k = (k_1,\cdots ,k_d)\in \mathbb {Z}^d, \end{aligned}$$

is not only orthonormal in the continuous space $L^2(\mathbb {T}^d)$, but (‘modulo *m*’) also with respect to the discrete inner product $\langle \cdot ,\cdot \rangle _{n}$, i.e.

13 $$\begin{aligned} \langle e_j,e_k \rangle _n = \left\{ \begin{array}{l} 1,\quad \text {if } j_l \equiv k_l \mod m, \qquad \forall l \in \{1,\cdots ,d\},\\ 0,\quad \text {otherwise.} \end{array}\right. \end{aligned}$$

The scale of isotropic Sobolev spaces $H_{s}(\mathbb {T}^d)$ is defined in terms of the Fourier coefficients $f_k = \int _{\mathbb {T}^d} f(x) e_k(x)\, dx$ of functions $f\in L^2(\mathbb {T}^d)$, as (for |*k*| any norm on $\mathbb {R}^{d}$)

$$\begin{aligned} H_{s}(\mathbb {T}^d) := \Bigl \{ f\in L^2(\mathbb {T}^d): \ \Vert f\Vert _{H_s}^2:=\sum \limits _{k\in \mathbb {Z}^d} (1 + |k|)^{2s} |f_k|^2 <\infty \Bigr \}. \end{aligned}$$

For smoothness levels $s\in \mathbb {N}$, this norm is equivalent to the canonical Sobolev norm $\sum _{|l|_1 \le s} \Vert D^l f \Vert _{L^2(\mathbb {T}^d)}$.

The spaces $V_j$ obtained as the linear span of the basis elements, ordered suitably, satisfy Assumption 2.2. Due to (13), the space $\widetilde{W}_n = \textsf{Span} \{e_k: |k|_\infty \le (m-1)/2 \}$ satisfies (10) with $C_1 = C_2 = 1$.

As noted in Reiß (2008), the following estimates hold for $f\in H_{s}(\mathbb {T}^D)$,

$$\begin{aligned} \Vert f - Q_n f\Vert _{L^2}{} & {} \lesssim n^{-s/d} \Vert f\Vert _{H_{s}},\\ \Vert Q_n f - \mathcal {I}_n f\Vert _{}{} & {} \lesssim _d n^{-s/d} \Vert f\Vert _{H_{s}}. \qquad \text { if } s>d/2. \end{aligned}$$

Here $Q_n$ is the orthogonal projection on $\widetilde{W}_n$. Consequently (11) is fulfilled for $s > s_d=d/2$.

#### Example 2.11

(Wavelets). This example is adapted from Section 3.3 in Reiß (2008). Let $\mathcal {D} = \mathbb {T}^d = (0,1]^d$, for $d\in \mathbb {N}$, and consider the design points $\{k 2^{-j}\}_{k\in \{1,\cdots ,2^j\}^d}$, where $n = 2^{jd}$ for some $j\in \mathbb {N}$. We consider a multi-resolution analysis $\{V_j\}_{j\ge 0}$ on $L^2(\mathbb {T}^d)$ obtained by periodisation and tensor products. Let $\widetilde{\phi }$ be a standard orthonormal scaling function of an *S*-regular multi-resolution analysis for $L^2(\mathbb {R})$, with compact support in $[S-1,S]$. In particular, the polynomial exactness condition is satisfied: $\sum _{k\in \mathbb {Z}} k^q \widetilde{\phi }(x - k) - x^q$ is a polynomial of maximal degree $q-1$ for $q\in [0,S -1]$. As shown in Reiß (2008), the functions

$$\begin{aligned} e_{j,k} (x_1,\ldots , x_d)= \sum \limits _{m\in \mathbb {Z}^d} 2^{jd/2}\prod _{i=1}^d\widetilde{\phi }(2^j x_i - k_i +2^j m_i), \end{aligned}$$

are well defined and form an orthonormal basis in $L^2(\mathbb {T}^d)$. Furthermore, for $\widetilde{W}_{n} := V_j = \textsf{Span}\{e_{j,k}\ |\ k\in \{1,\cdots ,2^j\}^d \}$ with $n=2^{jd}\ge 2S - 1$, conditions (10) is satisfied with constants $C_1,C_2$ that depend only on $\widetilde{\phi }$. Moreover, for the functions $e_{j,k}$ belong to the Besov space $B_{2,2}^s(\mathbb {T^d})$, for $s<S$, and, for every *f* in this Besov space and $d/2<s< S$,

$$\begin{aligned} \Vert f - \mathcal {I}_n f\Vert _{L^2} \lesssim n^{-s/d} \Vert f\Vert _{B_{2,2}^s}. \end{aligned}$$

Thus (11) is satisfied, with the smoothness scale $(H_s)_{s\in \mathbb {R}}$ taken equal to the canonical Sobolev spaces (i.e. Besov spaces $B_{2,2}^s$) on $\mathbb {T}^d$.

Other examples of suitable discretisation spaces are provided by orthogonal polynomials, for instance the systems of Legendre, Chebyshev, or Jacobi polynomials, etc., for suitably chosen design points. Canonical Sobolev spaces on $\mathbb {T} = (0,1]$ are Hilbert scales, and Assumption 2.2 is satisfied by standard Sturm-Liouville theory (see 5.2 in Canuto et al. (2010)): the polynomials form infinitely differentiable orthogonal bases in $L^2(\mathbb {T})$. Assumption 2.7 is satisfied with Gaussian quadrature points as design points (Section 5.3 in Canuto et al. (2010)). These results can be extended to multivariate domains by using tensor products.

## General Contraction Rates

In this section we present two general theorems on posterior contraction, which we apply to different priors in later sections. The theorems address the situation of discrete observations. After making the appropriate modifications, their proofs borrow from the proofs for the continuous case in Gugushvili et al. (2020).

We form the posterior distribution ${\mathrm {\Pi }}_n ( \cdot \,|\ Y^n )$ as in (3), given a prior ${\mathrm {\Pi }}$ on the space $H=H_0$ and an observation $Y^n=(Y_1,\ldots , Y_n)$, whose conditional distribution given *f* is determined by the model (1). We study this random distribution under the assumption that $Y^n$ follows the model (1) for a given ‘true’ function $f=f_0$, which we assume to be an element of $H_{\beta }$ in a given smoothness scale $(H_s)_{s\in \mathbb {R}}$, as in Definition 2.1.

The theorem is stated in terms of the Galerkin solution to the continuous inverse problem, which is defined as follows. (See e.g., Kirsch (2011) for a general introduction to the Galerkin method and Appendix A for the two results needed in our proof.) Let $W_j=AV_j\subset G$ be the image under the operator *A* of a finite-dimensional approximation space $V_j$ linked to the smoothness scale $(H_s)_{s\in \mathbb {R}}$ as in Assumption 2.2, and let $Q_j: G\rightarrow W_j$ be the orthogonal projection onto $W_j$. The *Galerkin solution* to *Af* is the inverse image $f^{(j)}\in V_j$ of $Q_jAf$. The idea is to simplify *Af* by projection before inverting the map *A*. (A solution $f^{(j)}\in V_j$ to the equation $Af^{(j)}=Q_j Af$ exists, under our assumption that $A: H\rightarrow G$ is injective, which implies that *A* is a bijection between the finite-dimensional vector spaces $V_j$ and $W_j$.)

In our current setting we have no access to the continuous function *Af*, but only to its values at the design points. We overcome this by using the Galerkin solution $f^{(j,n)}=A^{-1}Q_j\mathcal {A}_nf$ to the interpolation $\mathcal {A}_nf$ of the discrete signal, where $\mathcal {A}_n = \mathcal {I}_n A$, for $\mathcal {I}_n$ the interpolation operator defined in Section 2.2. This *discrete Galerkin solution* is illustrated in the following diagramIn this scheme the space $\widetilde{W}_n$, used to construct the continuous interpolation, may or may not be equal to $W_n=A V_n$. Setting it equal to $W_n$ simplifies the scheme, but then the interpolation properties in Assumption 2.7 must be verified for $AV_n$.

### Theorem 3.1

For smoothness classes $(H_s)_{s\in \mathbb {R}}$ as in Definition 2.1, assume that $\Vert Af \Vert \simeq \Vert f\Vert _{-\gamma }$, for some $\gamma >0$. Let $f^{(j,n)}$ denote the discrete Galerkin solution to $\mathcal {A}_n f = \mathcal {I}_n Af$ relative to linear subspaces $V_j$ associated to $(H_s)_{s\in \mathbb {R}}$ as in Assumption 2.2 and interpolation spaces $\widetilde{W}_n$ satisfying (10)-(11). Let $f_0\in H_{\beta }$ and $Af_0\in G_{\beta +\gamma }$ for some $\beta \in (s_d-\gamma ,S_d-\gamma )$, and for $\eta _n\ge \varepsilon _{n}\downarrow 0$ such that $n\varepsilon _{n}^2\rightarrow \infty$, and $j_n\in \mathbb {N}$ such that $j_n\rightarrow \infty$, and some $c>0$, assume

14 $$\begin{aligned} j_n{} & {} \le c n\varepsilon _{n}^2,\end{aligned}$$

15 $$\begin{aligned} \eta _n{} & {} \ge \frac{\varepsilon _{n}}{\delta (j_n,\gamma )},\end{aligned}$$

16 $$\begin{aligned} \eta _n{} & {} \ge \delta (j_n,\beta ) \vee \frac{\delta _{d}(n, \beta +\gamma )}{ \delta (j_n, \gamma )}. \end{aligned}$$

Consider prior probability distributions ${\mathrm {\Pi }}$ on $H_0$ satisfying

17 $$\begin{aligned} {\mathrm {\Pi }}\bigl ( f\in H: \Vert A f - A f_0\Vert _n < \varepsilon _{n} \bigr ){} & {} \ge e^{-n\varepsilon _{n}^2}, \end{aligned}$$

18 $$\begin{aligned} {\mathrm {\Pi }}\bigl ( f\in H: \Vert f^{(j_n,n)}-f\Vert _{}> \eta _n\bigr ){} & {} \lesssim e^{-4n\varepsilon _{n}^2}. \end{aligned}$$

Then the posterior distribution in the model (1) contracts at the rate $\eta _n$ at $f_0$, i.e. for a sufficiently large constant *M* we have ${\mathrm {\Pi }}_n\bigl (f: \Vert f-f_0\Vert > M\eta _n\,|\ Y_1,\ldots , Y_n\bigr )\rightarrow 0$, in probability if $Y_1,\ldots , Y_n$ follow (1) with $f=f_0$.

### Proof

Let $p_f^{(n)}$ be the (multivariate-normal) density of $Y^n$. The Kullback-Leibler divergence $P_{f_0}^{(n)}\log (p_{f_0}^{(n)}/p_f^{(n)})$ and variation $P_{f_0}^{(n)}\bigl (\log (p_{f_0}^{(n)}/p_f^{(n)})\bigr ){}^2$ can be computed to be $n\Vert A f-A f_0\Vert _n^2/2$ and twice this quantity, respectively. Therefore, by (17) the set where these quantities are bounded above by $n\varepsilon _{n}^2$ has prior probability at least $e^{-n\varepsilon _{n}^2}$.

It follows by Lemma 8.2 of Ghosal and van der Vaart (2017) that the events $B_n:=\{\int p_f^{(n)}/p_{f_0}^{(n)}(y^n)\,d{\mathrm {\Pi }}(f)\ge e^{-2n\varepsilon _{n}^2}\}$ satisfy $P_{f_0}^{(n)}(B_n)\rightarrow 1$.

Furthermore, by (18) and Theorem 8.20 in the same reference, the posterior probability ${\mathrm {\Pi }}_n\bigl (\Vert f^{(j_n,n)}-f\Vert >\eta _n\,|\ Y^n\bigr )$ tends to zero in $P_{f_0}^{(n)}$ probability.

Let $\mathcal {F}_n=\{f: \Vert f-f_0\Vert >M\eta _n,\Vert f^{(j_n,n)}-f\Vert \le \eta _n\}$. We show below that there exist tests $\tau _{n}$ such that $P_{f_0}^{(n)}\tau _{n} \rightarrow 0$ and $P_f^{(n)}(1-\tau _{n})\le e^{-4n\varepsilon _{n}^2}$, for every $f\in \mathcal {F}_n$. Then to prove the assertion of the theorem, we bound ${\mathrm {\Pi }}_n\bigl (f: \Vert f-f_0\Vert > M \eta _n\,|\ Y_1,\ldots ,Y_n\bigr )$ by

$$\begin{aligned} \tau _{n}+ 1_{B_n^c}+{\mathrm {\Pi }}_n\bigl (\Vert f^{(j_n,n)}-f\Vert > \eta _n\,|\ Y^n\bigr ) +{\mathrm {\Pi }}_n(\mathcal {F}_n\,|\ Y^n)1_{B_n}(1-\tau _{n}). \end{aligned}$$

The expectations under $P_{f_0}^{(n)}$ of the first three terms tend to zero by the preceding. In the fourth term we bound the denominator in Bayes’s formula (3) divided by the likelihood at $f_0$ by $e^{-2n\varepsilon _{n}^2}$, and next bound its expectation by

$$\begin{aligned} e^{2n\varepsilon _{n}^2} P_{f_0}^{(n)}\int _{\mathcal {F}_n} \frac{p_f^{(n)}}{p_{f_0}^{(n)}}\,d{\mathrm {\Pi }}(f)(1-\tau _{n}) \le e^{2n\varepsilon _{n}^2} \sup _{f\in \mathcal {F}_n}P_f^{(n)}(1-\tau _{n})\le e^{-2n\varepsilon _{n}^2}\rightarrow 0. \end{aligned}$$

This proof of the theorem can now be completed by constructing the tests $\tau _{n}$.

Define the operator $R_j: G\mapsto V_j$ by $R_j=A^{-1}Q_j$, where $Q_j:G\rightarrow W_j$ is the orthogonal projection onto $W_j=AV_j$ and $A^{-1}$ is the inverse of *A* (restricted to $W_j$). Consider the tests defined by, for a given constant $M_0$ determined later and $Y^{(n)}$ defined in (12),

19 $$\begin{aligned} \tau _n=1\bigl \{\Vert R_{j_n}Y^{(n)}-f_0\Vert \ge M_0 \eta _n\bigr \}. \end{aligned}$$

Since by definition $f^{(j,n)}=R_j\mathcal {A}_n f$, we have

20 $$\begin{aligned} R_j Y^{(n)}=R_j \mathcal {A}_n f+\frac{1}{\sqrt{n}}R_j \xi ^{(n)}=f^{(j,n)}+\frac{1}{\sqrt{n}}R_j \xi ^{(n)}. \end{aligned}$$

The variable $R_j \xi ^{(n)}=R_j Q_j\xi ^{(n)}$ is a centred Gaussian random element in $V_j$ with strong and weak second moments

$$\begin{aligned} \textrm{E}\bigl \Vert R_j \xi ^{(n)}\bigr \Vert ^2{} & {} \le \Vert R_j\Vert ^{2} \textrm{E}\Vert Q_j\xi ^{(n)}\Vert ^2 \lesssim \Vert R_j\Vert ^2j\lesssim \frac{j}{\delta (j,\gamma )^2},\\ \sup _{\Vert f\Vert \le 1} \textrm{E} \langle R_j \xi ^{(n)}, f\rangle ^2{} & {} \!=\!\sup _{\Vert f\Vert \le 1} \!\textrm{E} \langle \xi ^{(n)}, R_j^*f\rangle _G^2 \lesssim \! \sup _{\Vert f\Vert \le 1}\Vert R_j^*f\Vert _G^2\!\le \! \Vert R_j^*\Vert ^2\!\lesssim \! \frac{1}{\delta (j,\gamma )^2}. \end{aligned}$$

In both cases the inequality on $\Vert R_j\Vert =\Vert R_j^*\Vert$ at the far right side follows from (A3A4), and we also use that, by Lemma 2.8, the covariance operator of $\xi ^{(n)}$ is bounded above by a multiple of the projection onto $\widetilde{W}_n$, and hence the identity, so that the covariance operator of $Q_j \xi ^{(n)}$ is bounded above by a multiple of $Q_j$.

The first inequality shows that the first moment $\textrm{E}\Vert R_j \xi ^{(n)}\Vert$ of the variable $\Vert R_j\xi ^{(n)}\Vert$ is bounded above by $\sqrt{j}/\delta (j,\gamma )$. By Borell’s inequality (e.g. Lemma 3.1 in Ledoux and Talagrand (1991) and subsequent discussion), applied to the Gaussian random variable $R_j\xi ^{(n)}$ in $H_0$, we see that, there exist positive constants *a* and *b* such that, for every $t>0$,

$$\begin{aligned} \Pr \Bigl (\Vert R_j\xi ^{(n)}\Vert >t+a\frac{\sqrt{j}}{\delta (j,\gamma )}\Bigr )\le e^{-bt^2\delta (j,\gamma )^2}. \end{aligned}$$

For $t=2\sqrt{n} \eta _n/\sqrt{b}$ and $\eta _n$, $\varepsilon _{n}$ and $j_n$ satisfying (14), (15) and (16) this yields, for some $a_1>0$,

21 $$\begin{aligned} \Pr \Bigl (\Vert R_{j_n}\xi ^{(n)}\Vert >a_1\sqrt{n}\eta _n\Bigr )\le e^{-4n\varepsilon _{n}^2}. \end{aligned}$$

We apply this to bound the two error probabilities of the tests $\tau _{n}$.

Under $f_0$ the decomposition (20) is valid with $f=f_0$, and hence $R_jY^{(n)}-f_0=n^{-1/2}R_j\xi ^{(n)}+f_0^{(j,n)}-f_0$. By the triangle inequality it follows that $\tau _{n}=1$ implies that $n^{-1/2}\Vert R_{j_n}\xi ^{(n)}\Vert \ge M_0\eta _n-\Vert f_0^{(j,n)}-f_0\Vert$. By the triangle inequality followed by (A3A4) and (11), and (A5),

$$\begin{aligned} \Vert f_0^{(j,n)}-f_0\Vert \le{} & {} \Vert R_j\Vert \,\Vert \mathcal {I}_{n} A f_0- Af_0 \Vert _{}+ \Vert R_j Af_0 - f_0\Vert _{}\\ \lesssim{} & {} \frac{\delta _d(n, \beta +\gamma )}{\delta (j_n, \gamma )}\Vert Af_0\Vert _{\beta +\gamma } + \delta (j_n, \beta )\Vert f_0\Vert _{\beta } \le M_1 \eta _n, \end{aligned}$$

by assumption (16). Hence the probability of an error of the first kind satisfies

$$\begin{aligned} P_{f_0}^{(n)} \tau _{n} \le \Pr \Bigl (\frac{1}{\sqrt{n}}\Vert R_{j_n}\xi ^{(n)}\Vert \ge (M_0-M_1)\eta _n\Bigr ), \end{aligned}$$

For $M_0-M_1>a_1$, the right side is bounded by $e^{-4n\varepsilon _{n}^2}$, by (21).

Under *f* the decomposition (20) gives that $R_jY^{(n)}-f_0=n^{-1/2}R_j\xi ^{(n)}+f^{(j,n)}-f_0$. By the triangle inequality $\tau _{n}=0$ implies that $n^{-1/2}\Vert R_{j_n}\xi ^{(n)}\Vert \ge \Vert f^{(j_n,n)}-f_0\Vert - M_0\eta _n$. For *f* such that $\Vert f-f_0\Vert >M\eta _n$ and $\Vert f-f^{(j_n,n)}\Vert \le \eta _n$, we have $\Vert f^{(j_n,n)}-f_0\Vert \ge (M-1)\eta _n$. Hence the probability of an error of the second kind satisfies

$$\begin{aligned} P_{f}^{(n)}(1- \tau _n) \le \Pr \Bigl (\frac{1}{\sqrt{n}}\Vert R_{j_n}\xi ^{(n)}\Vert \ge (M-1-M_0)\eta _n\Bigr ), \end{aligned}$$

For $M-1-M_0>a_1$, this is bounded by $e^{-4n\varepsilon _{n}^2}$, by (21).

We can first choose $M_0$ large enough so that $M_0-M_1>a_1$, and next *M* large enough so that $M-1-M_0>a_1$, to finish the proof.

Inequality (17) is the usual *prior mass condition* for the ‘direct problem’ of estimating *Af* at the design points (see Ghosal et al. (2000)). It determines the rate of contraction $\varepsilon _{n}$ of the posterior distribution of *Af* to $Af_0$ relative to the discrete seminorm $\Vert \cdot \Vert _n$. The rate of contraction $\eta _n$ of the posterior distribution of *f* is slower due to the necessity of (implicitly) inverting the operator *A*. The theorem shows that the rate $\eta _n$ depends on the combination of the prior, through (18), and the inverse problem, through the various approximation rates.

The theorem has a similar form as the theorem obtained in Gugushvili et al. (2020) in the case of observation of a continuous signal in white noise. Presently the Galerkin projection $f^{(j,n)}$ in (18) incorporates the errors of both inversion and discretisation. The factor $\delta _d(n, \beta +\gamma )/\delta (j_n,\gamma )$ in (16), which arises from having discrete observations only, will typically be negligible relative to $\delta (j_n, \beta )$.

The following refinement of the preceding theorem is appropriate for certain priors that are constructed to give an optimal contraction rate for multiple values of the smoothness $\beta$ of the true parameter simultaneously. It considers a mixture prior of the form

22 $$\begin{aligned} {\mathrm {\Pi }}=\int {\mathrm {\Pi }}_{\tau }\,dQ(\tau ), \end{aligned}$$

where ${\mathrm {\Pi }}_{\tau }$ is a prior on *H*, for every given ‘hyper-parameter’ $\tau$ running through some measurable space, and *Q* is a prior on this hyper-parameter. The idea is to adapt the prior to multiple smoothness levels through the hyper-parameter $\tau$. The proof is very similar to the proof of Theorem 3.3 in Gugushvili et al. (2020) and is omitted.

### Theorem 3.2

Consider the setup and assumptions of Theorem 3.1 with a prior of the form (22). Assume that (14), (15), (16) and (17) hold, but replace (18) by the pair of conditions, for numbers $\eta _{n,\tau }$ and $C>0$ and every $\tau$,

23 $$\begin{aligned} {\mathrm {\Pi }}_{\tau }\bigl (f: \Vert f^{(j_n,n)}-f\Vert > \eta _{n,\tau }\bigr ){} & {} \le e^{-4n\varepsilon _{n}^2}, \end{aligned}$$

24 $$\begin{aligned} {\mathrm {\Pi }}_{\tau }\bigl (f: \Vert f-f_0\Vert <2\eta _{n,\tau }\bigr ){} & {} \le e^{-4n\varepsilon _{n}^2}, \quad \forall \tau \text { with }\eta _{n,\tau }\ge C\eta _n. \end{aligned}$$

Then the posterior distribution in the model (1) contracts at the rate $\eta _n$ at $f_0$, i.e. for a sufficiently large constant *M* we have ${\mathrm {\Pi }}_n\bigl (f: \Vert f-f_0\Vert > M\eta _n\,|\ Y_1,\ldots , Y_n\bigr )\rightarrow 0$, in probability if $Y_1,\ldots , Y_n$ follow (1) with $f=f_0$.

## Random Series Priors

Suppose that $\{\phi _i\}_{i\in \mathbb {N}}$ is an orthonormal basis of $H=H_0$ that gives optimal approximation relative to the scale of smoothness classes $(H_s)_{s\in \mathbb {R}}$ in the sense that the linear spaces $V_j = \textsf{Span}\{\phi _i\}_{i<j}$ satisfy Assumption 2.2. Consider a prior defined as the law of the random series

25 $$\begin{aligned} f= \sum \limits _{i=1}^M f_i \phi _i, \end{aligned}$$

where *M* is a random variable in $\mathbb {N}$ independent from the independent random variables $f_1,f_2,\ldots$ in $\mathbb {R}$.

### Assumption 4.1

(Random series prior).

1. The probability density function $p_M$ of *M* satisfies, for some positive constants $b_1,b_2$,
  $$\begin{aligned} e^{-b_1 k}\lesssim p_M(k) \lesssim e^{-b_2 k}, \qquad \forall k\in \mathbb {N}. \end{aligned}$$
2. The variable $f_i$ has density $p(\cdot /\kappa _i)/\kappa _i$, for a given Lebesgue probability density *p* on $\mathbb {R}$ and a positive constant $\kappa _i$ such that, for some $C>0$ and $0<v<w<\infty$, $\beta _{0}>0$ and $\alpha >0$,
  26 $$\begin{aligned} e^{-C|x|^{w}}{} & {} \lesssim p(x) \lesssim e^{-C|x|^v},\end{aligned}$$
  27 $$\begin{aligned} i^{-\beta _{0}/d}{} & {} \lesssim \kappa _i \lesssim i^{\alpha }. \end{aligned}$$

Priors of this type were studied in Arbel et al. (2013), and applied to inverse problems in the SVD framework in Ray (2013). The assumptions on $p_M$, *p* and the scale parameters $\kappa _i$ are mild and satisfied by many examples. For Gaussian variables $f_j$ and degenerate *M*, the series (25) is a Gaussian process.

The basis functions are connected to the operator *A*, because they must generate the smoothness scale such that Assumption 2.5 holds, but this allows considerable freedom. For instance, a basis on wavelets may be appropriate for differential operators that are smoothing in the Sobolev scale.

### Theorem 4.2

(Random Series Prior). Let $(\phi _i)_{i\in \mathbb {N}}$ be an orthonormal basis of $H_0$ such that the spaces $V_j = \textsf{Span}\{\phi _i\}_{i<j}$ satisfy Assumption 2.2 with $\delta (j,s) = j^{-s/d}$ relative to smoothness classes $(H_s)_{s\in \mathbb {R}}$ as in Definition 2.1. Assume that Assumption 2.7 holds with $\delta _d(n,s)=n^{-s/d}$ and sufficiently large $S_d$. Suppose Assumption 2.5 holds for the operator *A*, and $f_0\in H_{\beta }$ for some $\beta \in (0,S]$ such that $\beta +\gamma >s_d\vee d/2$. Then, for the random series prior defined in (25) and satisfying Assumption 4.1 with $\beta _0 \le \beta$, and sufficiently large $M>0$, for $\tau =(\beta + \gamma )(1+2\gamma /d)/(2\beta + 2 \gamma + d)$,

$$\begin{aligned} {\mathrm {\Pi }}_n\Bigl ( f: \Vert f - f_0\Vert _{} > M n^{-\beta /(2\beta + 2 \gamma + d)} (\log n)^{\tau } \,|\ Y^{(n)}\Bigr ) \overset{\mathbb {P}^{(n)}_{f_0} }{\rightarrow } 0. \end{aligned}$$

The rate $n^{-\beta /(2\beta + 2 \gamma + d)}$ is known to be the minimax rate of estimation of a $\beta$-regular function on a *d*-dimensional domain, in an inverse problem with inverse parameter $\gamma$ (see, e.g., Cohen et al. (2004)). The assumption that $\delta (j,s) = j^{-s/d}$ places the setup of the theorem in this setting, and hence the rate of contraction obtained in the preceding theorem is the minimax rate up to a logarithmic factor. The rate is adaptive to the regularity of $\beta$ of the true parameter, which is not used in the construction of the prior, apart from the assumption that $\beta \ge \beta _0$. (See Ghosal et al. (2008) and Chapter 10 in Ghosal and van der Vaart (2017) for general discussion of adaptation in the Bayesian sense.)

The proof of the theorem is deferred to Section 6; it will be based on Theorem 3.1.

## Gaussian Priors and Gaussian Mixtures

If the function *f* in (1) is equipped with a Gaussian prior, then the corresponding posterior distribution is Gaussian as well. The posterior mean is then equal to the solution found by the method of Tikhonov-type regularization (see e.g.  Knapik et al. (2011); Florens and Simoni (2016); Stuart (2010)). Because a Gaussian prior possesses a fixed smoothness level, it is usually scaled with a hyper-parameter, leading to a mixture of Gaussian priors. In this section we derive a rate of contraction both for Gaussian priors of fixed smoothness and for scaled Gaussian priors.

By definition a random variable *F* with values in a Hilbert space $H_0$ is Gaussian if $\langle F, g\rangle _{}$ is normally distributed, for every $g\in H_0$. It is *centred* if these variables have zero means, and it has *covariance operator* $C: H_0\rightarrow H_0$ if $\textrm{E} \langle F, g\rangle _{}^2=\langle C g,g\rangle _{}$, for every $g\in H_0$.

A Gaussian prior can also, and without loss of generality, be described through a basis expansion. Given an orthonormal basis $(\phi _j)_{j\in \mathbb {N}}$ of $H_0$ and a sequence of independent variables $F_j\sim N(0, \sigma _{j}^2)$, for given $\sigma _{j}^2>0$ with $\sum _j \sigma _{j}^2<\infty$, the variable $F=\sum _{j=1}^\infty F_j\phi _j$ is Gaussian with covariance operator $C_{\sigma } g=\sum _j \sigma _{j}^2g_j\phi _j$ if $g=\sum _j g_j\phi _j$.

It is customary to relate such a prior to the generator of a *Hilbert scale* of spaces, as follows. We restrict to the case that $\sigma _{j}$ is polynomial in 1/*j*. We define spaces $H_s$ as in Example 2.3, with $b_j=j^{1/d}$. The operator $Lg=\sum _j b_jg_j\phi _j$ is an isometry $L: H_s\rightarrow H_{s-1}$, for every *s*, and is said to generate the Hilbert scale $(H_s)_{s\in \mathbb {R}}$. For $\alpha >d/2$, the operator $L^{-2\alpha }$ has finite trace $\sum _j j^{-2\alpha /d}$ and defines a Gaussian distribution on $H_0$. Because $L^sF$ has covariance operator $L^{2s-2\alpha }$, which has finite trace if $s<\alpha -d/2$, this variable is a proper Gaussian variable in $H_0$, for every $s<\alpha -d/2$. Hence *F* is a proper Gaussian variable in $H_s=L^{-s}H_0$ for the same values of *s*. We may describe this by saying that the prior with covariance operator $L^{-2\alpha }$ is ‘(nearly) of smoothness $\alpha -d/2$’.

The following theorem gives a posterior contraction rate for this prior. The proof is deferred to Section 6.

### Theorem 5.1

(Gaussian Prior). Suppose Assumption 2.5 holds for the operator *A* relative to the Hilbert scale $(H_s)_{s\in \mathbb {R}}$ generated by the operator $L^{-1}$, as indicated. Assume that Assumption 2.7 holds with $\delta _d(n,s)=n^{-s/d}$ and $s_d=d/2$. Assume $f_0 \in H_{\beta }$, for some $\beta >0$ such that $\beta + \gamma > d/2$, and let the prior be zero-mean Gaussian with covariance operator $L^{-2\alpha }$, for some $\alpha >d/2\vee \beta$ such that $\alpha +\gamma >d$. Then the posterior distribution satisfies, for sufficiently large $M>0$,

$$\begin{aligned} {\mathrm {\Pi }}_n\Bigl ( f: \Vert f - f_0\Vert _{} > M n^{-((\alpha - d/2)\wedge \beta )/(2\alpha +2\gamma )} \,|\ Y^{(n)}\Bigr ) \overset{\mathbb {P}^{(n)}_{f_0} }{\rightarrow } 0. \end{aligned}$$

The Gaussian prior in the preceding theorem has a fixed smoothness $\alpha -d/2$, which results in a contraction rate that depends on both the prior smoothness and the smoothness $\beta$ of the true function. This rate is equal to the minimax rate $n^{-\beta /(2\beta + 2\gamma +d)}$ (see Cohen et al. (2004)) only when $\alpha - d/2 = \beta$, i.e., when the prior smoothness $\alpha -d/2$ matches the true smoothness $\beta$. By mixing over Gaussian priors of varying smoothness, the minimax rate can often be obtained simultaneously for a range of values $\beta$ (cf. Knapik et al. (2016), van der Vaart (2010), Szabó et al. (2013)).

In the following theorem we consider mixtures of the mean-zero Gaussian priors with covariance operators $\tau ^{2}L^{-2\alpha }$ over the ‘hyper-parameter’ $\tau$. Thus the prior ${\mathrm {\Pi }}$ is the distribution of $\tau F$, where *F* is a zero-mean Gaussian variable in $H_0$ with covariance operator $L^{-2\alpha }$ as before, and $\tau$ is an independent scale parameter. The variable $1/\tau ^{a}$ may be taken to possess a Gamma distribution for some given $0<a\le 2$, or, more generally, should satisfy the following mild condition.

### Assumption 5.2

The distribution *Q* of $\tau$ has support $[0,\infty )$ and satisfies

$$\begin{aligned} \left\{ \begin{array}{ll} -\log Q\bigl ((t,2t)\bigr )\lesssim t^{-2}, &{} \text { as } t \downarrow 0,\\ -\log Q\bigl ((t,2t)\bigr )\lesssim t^{d/(\alpha -d/2)}, &{} \text { as } t \rightarrow \infty . \end{array}\right. \end{aligned}$$

### Theorem 5.3

(Gaussian mixture prior). Suppose Assumption 2.5 holds for the operator *A* relative to the Hilbert scale $(H_s)_{s\in \mathbb {R}}$ generated by the operator $L^{-1}$, as indicated. Assume that Assumption 2.7 holds with $\delta _d(n,s)=n^{-s/d}$ and $s_d=d/2$. Assume that $f_0 \in H_{\beta }$, for some $\beta >0$ such that $\beta +\gamma >d/2$, and let the prior be a mixture of the zero-mean Gaussian $\tau ^{2} L^{-2\alpha }$ over the parameters $\tau$ equipped with a prior satisfying Assumption 5.2, for some $\alpha >d/2$ such that $\alpha +\gamma >d$. Then the posterior distribution satisfies, for sufficiently large $M>0$,

$$\begin{aligned} {\mathrm {\Pi }}_n\Bigl ( f: \Vert f - f_0\Vert _{} > M n^{-\beta /(2\beta + 2\gamma + d)} \,|\ Y^{(n)}\Bigr ) \overset{\mathbb {P}^{(n)}_{f_0} }{\rightarrow } 0. \end{aligned}$$

The proof of the theorem is deferred to Section 6.

## Proofs

### Proof of Theorem 4.2

The theorem is a corollary to Theorem 3.1. We shall verify the conditions with

$$\begin{aligned} \varepsilon _{n} \simeq (\log n/n)^{(\beta + \gamma )/(2 \beta + 2 \gamma +d)}, \quad j_n \simeq n^{d/(2\beta +2\gamma +d)}(\log n)^{(2\beta +2\gamma )/(2\beta +2\gamma +d)}. \end{aligned}$$

Let $P_j$ be the orthogonal projection of *H* on the linear span of the first $j-1$ basis elements $\phi _j$, and define an additional sequence of integers by

$$\begin{aligned} i_n\simeq (n/\log n)^{d/(2\beta +2\gamma +d)}. \end{aligned}$$

By the orthogonality of the basis $(\phi _i)$, the function $\phi _j$ is orthogonal to the space $V_j$ spanned by $(\phi _i)_{i<j}$. Hence $P_j\phi _j=0$, so that $\Vert \phi _j\Vert _{-s}\le \delta (j,s)\Vert \phi _j\Vert _{}\lesssim j^{-s/d}$, for every *j* and $s\ge 0$, by (A1). The same estimate is also true for negative smoothness levels *s* such that $0<-s<S$, directly by assumption (7). Therefore, by the triangle inequality, we have

$$\begin{aligned} \Vert f\Vert _s\lesssim \sum \limits _j |f_j| j^{s/d},\qquad \text { if } f=\sum \limits _jf_j\phi _j. \end{aligned}$$

Furthermore, since $f_0\in H_{\beta }$ by assumption, the norm duality (5) gives that $|f_{0,i}| = |\langle f_0,\phi _i \rangle _{}|\le \Vert f_0\Vert _{\beta } \Vert \phi _i\Vert _{-\beta } \lesssim i^{-\beta /d}$.

First we verify the prior condition (17) of the direct problem. By Lemma 2.9, $\Vert Af - Af_0\Vert _n \simeq \Vert \mathcal {I}_n(Af - Af_0)\Vert$. By several applications of the triangle inequality, since $\beta +\gamma \in (s_d,S_d)$, $\Vert Af\Vert _{\beta +\gamma }\simeq \Vert f\Vert _{\beta }$ and $\Vert Af-Af_0\Vert \simeq \Vert f-f_0\Vert _{-\gamma }$,

$$\begin{aligned} \Vert \mathcal {I}_n(Af -{} & {} Af_0)\Vert _{} \le \Vert \mathcal {I}_n Af - Af \Vert + \Vert \mathcal {I}_nAf_0 - Af_0\Vert +\Vert Af - Af_0 \Vert _{}\\{} & {} \lesssim \delta _{d}(n,\beta +\gamma )\bigl (\Vert f\Vert _{\beta } +\Vert f_0\Vert _{\beta }\bigr )+\Vert f - P_{i_n}f_0\Vert _{-\gamma } + \Vert f_0 - P_{i_n}f_0\Vert _{-\gamma }\\{} & {} \lesssim \delta _{d}(n,\beta +\gamma ) \Vert f - P_{i_n}f_0\Vert _{\beta } +\Vert f - P_{i_n}f_0\Vert _{-\gamma }\\{} & {} \qquad +\delta _{d}(n,\beta +\gamma )\bigl (\Vert P_{i_n}f_0\Vert _{\beta } +\Vert f_0\Vert _{\beta }\bigr )+ \delta (i_n,\gamma ) \delta (i_n,\beta )\Vert f_0\Vert _{\beta }, \end{aligned}$$

by (A1). The last term is of the order $\delta (i_n,\gamma )\delta (i_n,\beta )=i_n^{-(\gamma + \beta )/d}\simeq \varepsilon _{n}$, while the second last term is bounded above by $\delta _{d}(n,\beta +\gamma ) \bigl (\Vert f_0\Vert _{}/\delta (i_n,\beta )+\Vert f_0\Vert _{\beta } \bigr )\ll \varepsilon _{n}$, if $(\beta +\gamma )^2> \beta d/2$. For $f = \sum _{i=1}^{i_n-1}f_i \phi _i \in V_{i_n}$, the sum of the first two terms on the right is bounded above by

$$\begin{aligned} \delta _{d}(n,\beta +\gamma )\sum \limits _{i=1}^{i_n-1}|f_i-f_{0,i}| i^{\beta /d}+ \sum \limits _{i=1}^{i_n-1}|f_i - f_{0,i}| i^{-\gamma /d} \lesssim \sum \limits _{i=1}^{i_n-1}|f_i - f_{0,i}| i^{-\gamma /d}. \end{aligned}$$

By arguments as in Ray (2013), it is shown in Section 8.1 of Gugushvili et al. (2020) that the right side of this equation is bounded above by $\varepsilon _{n}$ with prior probability at least $e^{-n\varepsilon _{n}^2/2}$. Since also ${\mathrm {\Pi }}(M=i_n-1)\ge e^{-b_1i_n}\ge e^{-n\varepsilon _{n}^2/2}$, it follows that (17) is satisfied.

Next we verify (18). Since ${\mathrm {\Pi }}(M\ge j_n)\le e^{-b_2'j_n}\le e^{-4n\varepsilon _{n}^2}$, by Assumption 4.1, we may intersect the event in (18) with the event $M<j_n$. For $M<j$ the random series $f=\sum _{i=1}^Mf_i\phi _i$ is contained in $V_j$ and the Galerkin approximation $R_j Af$ of *f* is exact. Since $f^{(j,n)}=R_j\mathcal {I}_n Af$, the triangle inequality followed by (A3A4) and (11) give, for $s+\gamma \in (s_d , S_d)$ and $f\in V_j$, in view of (7),

$$\begin{aligned} \Vert f^{(j,n)}-f\Vert{} & {} \le \Vert R_j\mathcal {I}_n Af-R_jAf\Vert +\Vert R_j Af - f\Vert \\{} & {} \le \frac{\delta _d(n,s+\gamma )}{\delta (j,\gamma )} \Vert f\Vert _{s}+0 \le \frac{\delta _{d}(n,s+\gamma )}{\delta (j,\gamma )\delta (j,s)} \Vert f\Vert . \end{aligned}$$

We conclude that it suffices to prove that

28 $$\begin{aligned} {\mathrm {\Pi }}\Bigl (\sum \limits _{i=1}^{j_n-1}f_i^2 > \eta _n^2 \Bigl (\frac{n}{j_n}\Bigr )^{(2\gamma +2s)/d}\Bigr ) \le e^{-4n\varepsilon _{n}^2}. \end{aligned}$$

By Assumption 4.1 we have that

$$\begin{aligned} \textrm{E} \sum \limits _{i=1}^{j_n-1} f_i^2\simeq \sum \limits _{i=1}^{j_n-1} \kappa _i^2\lesssim j_n^{2\alpha +1}. \end{aligned}$$

$$\begin{aligned} \textrm{E} \Bigl |\sum \limits _{i=1}^{j_n-1} (f_i^{2} - \textrm{E} f_i^2)\Bigr |\lesssim \sqrt{\sum \limits _{i=1}^{j_n-1}\kappa _i^4}\lesssim j_n^{2\alpha +1/2}. \end{aligned}$$

Furthermore, by the tail bound on the density *p* we further have that the $\psi _{v/2}$ Orlicz norm of $f_i^2$ is bounded above by $\kappa _i^2$. Therefore, (see e.g. Proposition A.1.6 in van der Vaart and Wellner (1996)), for *q* conjugate to *v*/2,

$$\begin{aligned} \Bigl \Vert \sum \limits _{i=1}^{j_n-1} (f_i^{2} - \textrm{E} f_i^2)\Bigr \Vert _{\psi _{v/2}} \lesssim \left\{ \begin{array}{ll} j_n^{2\alpha +1/2}+(\log j_n)^{2/v}\max _{i< j_n} \kappa _i^2,&{}\text { if } v\le 2,\\ j_n^{2\alpha +1/2}+(\sum _{i<j_n} \kappa _i^{2q})^{1/q},&{}\text { if } 2<v\le 4, \end{array}\right. \end{aligned}$$

In both cases the first term $j_n^{2\alpha +1/2}$ dominates. Provided

$$\begin{aligned} \eta _n^2 (n/j_n)^{(2\gamma +2s)/d}=n^{(4(s+\gamma )(\beta +\gamma )/d-2\beta )/(2\beta +2\gamma +d)}(\log n)^a\gtrsim j_n^{2\alpha +1}, \end{aligned}$$

we can first centre $\sum _{i=1}^{j_n-1} f_i^2$ at mean zero, and next bound the tail of the centred variable with the help of the Orlicz norm. This will give a bound on the left side of (28) of the type

$$\begin{aligned} \frac{1}{\psi _{v/2}}\Bigl (\frac{n^{(4(s+\gamma )(\beta +\gamma )/d-2\beta )/(2\beta +2\gamma +d)}(\log n)^a}{j_n^{2\alpha +1/2}}\Bigr ). \end{aligned}$$

For sufficiently large *s* the argument is an arbitrarily large power of *n* times a logarithmic factor and hence this is easily smaller than $e^{-4n\varepsilon _{n}^2}$. Thus condition (18) holds provided *s*, which was required to belong to $(s_d-\gamma ,S_d-\gamma )$, can be chosen sufficiently large. This can be worked out to lead to the requirement that $2(\beta +\gamma )(S_d+\gamma )>d^2(v^{-1}\vee (\alpha +1/2))+\beta d$.

### Proof of Theorem 5.1

The theorem is a corollary to Theorem 3.1 with

29 $$\begin{aligned} \varepsilon _{n} \simeq n^{-(\beta \wedge (\alpha - d/2) + \gamma )/( 2\alpha + 2 \gamma )}. \end{aligned}$$

We verify that $\varepsilon _{n}$ satisfies the prior mass condition (17) of the direct problem, and next identify $\eta _n$ from the other prior mass condition (18).

In both cases we reduce the problem to the case of continuous observations by estimating the interpolation error, using the following lemma.

#### Lemma 6.1

Under the assumptions of Theorem 5.1, there exists a constant $c>0$ so that for $s<\alpha -d/2$ with $s+\gamma \in (s_d,S_d)$ and any $t>0$,

$$\begin{aligned} {\mathrm {\Pi }}\bigl (\Vert \mathcal {I}_nA f-Af\Vert _{}>t\bigr )\lesssim e^{-ct^2n^{(2s+2\gamma )/d}}. \end{aligned}$$

#### Proof

Since the prior of *f* is a centred Gaussian distribution with covariance operator $L^{-2\alpha }$, we have that $f\in H_s$, almost surely, for $s < \alpha -d/2$, and consequently, $Af \in G_{s+\gamma }$ almost surely. Assumptions (11) and (9) give

$$\begin{aligned} \Vert \mathcal {I}_nAf - Af\Vert _{}\lesssim \delta _{d}(n, s + \gamma ) \Vert Af\Vert _{s+\gamma }\simeq \delta _{d}(n, s + \gamma ) \Vert f\Vert _s = \delta _{d}(n, s + \gamma ) \Vert L^s f\Vert _{}. \end{aligned}$$

The variable $L^sF$, for $F\sim {\mathrm {\Pi }}$, possesses a centred Gaussian distribution in $H_0$ with covariance operator $L^{-2(\alpha - s)}$, which is finite trace since $\alpha -s>d/2$. Therefore, Borell’s inequality gives ${\mathrm {\Pi }}\bigl (\Vert L^sF\Vert _{}>t\bigr )\le e^{-ct^2}$, for every $t>0$ and a suitable constant $c>0$. Together with the preceding display, this implies the lemma.

For the proof of (17), we first note that $\Vert Af - Af_0\Vert _n \simeq \Vert \mathcal {I}_n(Af - Af_0)\Vert$, by Lemma 2.9. Next, by the triangle inequality,

30 $$\begin{aligned} \Vert \mathcal {I}_n(Af - Af_0)\Vert _{} \le \Vert \mathcal {I}_n Af - Af \Vert + \Vert \mathcal {I}_nAf_0 - Af_0\Vert _{}+ \Vert Af - Af_0\Vert . \end{aligned}$$

Since $Af_0\in G_{\beta +\gamma }$ and $\beta + \gamma > d/2 = s_d,$ the middle term satisfies $\Vert \mathcal {I}_nAf_0 - Af_0\Vert _{}\le \delta _{d}(n, \beta + \gamma ) \Vert Af_0\Vert _{\beta +\gamma } \simeq n^{-(\beta +\gamma )/d}\ll n^{-(\beta +\gamma )/(2\alpha +2\gamma )}\le \varepsilon _{n}$, if $\alpha +\gamma >d/2$, and hence is negligible in the context of (17).

Because $\alpha + \gamma > d$ by assumption, we have $d/2-\gamma <\alpha -d/2$. For *s* in the interval $(d/2-\gamma ,<\alpha -d/2)$, we have $s+\gamma >d/2=s_d$ and hence Lemma 6.1 gives that ${\mathrm {\Pi }}\bigl (\Vert \mathcal {I}_n Af - Af \Vert >d\varepsilon _{n}\bigr )$ is bounded above by $e^{-c_1\varepsilon _{n}^2n^{(2s+2\gamma )/d}}\ll e^{-n\varepsilon _{n}^2}$, since $s+\gamma >d/2$.

Using the inequality $\Pr (X+Y<\varepsilon )\ge \Pr (X<\varepsilon /2)-\Pr (Y>\varepsilon /2)$, for any random variables *X*, *Y*, and (30), we see the validity of (17) if ${\mathrm {\Pi }}\bigl ( \Vert Af - Af_0\Vert <\varepsilon _{n}\bigr )\ge e^{-n\varepsilon _{n}^2}$. This prior estimate is the same as the prior mass condition for the white noise model, which was studied in Gugushvili et al. (2020). The prior estimate follows from the following lemma, which is Lemma 9.2 from the latter paper.

#### Lemma 6.2

Under the assumptions of Theorem 5.1, for $f_0\in H_{\beta }$, as $\varepsilon \downarrow 0$,

$$\begin{aligned} -\log {\mathrm {\Pi }}\bigl (f: \Vert Af-Af_0\Vert _{}< \varepsilon \bigr ) \lesssim \left\{ \begin{array}{ll} \varepsilon ^{-d/(\alpha + \gamma -d/2)},&{}\text { if } d/2 <\alpha \le \beta + d/2, \\ \varepsilon ^{-(2\alpha - 2 \beta )/(\beta + \gamma )},&{}\text { if } \alpha > \beta + d/2. \end{array}\right. \end{aligned}$$

The next step of the proof is to bound the prior probability in (18), which involves the discrete Galerkin approximation $f^{(j,n)}=R_j\mathcal {I}_nAf$, where $R_j=A^{-1}Q_j$. By the triangle inequality,

31 $$\begin{aligned} \Vert f^{(j,n)}-f\Vert _{}{} & {} \le \Vert R_jAf-f\Vert _{}+\Vert R_j\mathcal {I}_nAf-R_jAf\Vert _{}\nonumber \\{} & {} \le \Vert f^{(j)}-f\Vert _{}+\frac{1}{\delta (j,\gamma )}\Vert \mathcal {I}_nAf-Af\Vert _{}, \end{aligned}$$

by (A3A4) and the definition of $f^{(j)}=R_jAf$. The contribution of the second term on the right to (18) can be bounded by ${\mathrm {\Pi }}\bigl (\Vert \mathcal {I}_nAf-Af\Vert _{}>\delta (j_n,\gamma )\eta _n\bigr ) \le {\mathrm {\Pi }}\bigl (\Vert \mathcal {I}_nAf-Af\Vert _{}>\varepsilon _{n}\bigr )$, because $\delta (j_n,\gamma )\eta _n\ge \varepsilon _{n}$, by (15). The last probability was seen to be $o(e^{-n\varepsilon _{n}^2})$ in the preceding. It follows that in the verification of (18) the discrete Galerkin solution $f^{(j_n,n)}$ may be replaced by the continuous Galerkin solution $f^{(j_n)}$. The prior estimates for this continuous case are given by the following lemma, which is Lemma 9.3 in Gugushvili et al. (2020). (In its proof $\Vert (R_jA-I)L^{-\alpha }\phi \Vert _0$ should be bounded by $\Vert L^{-\alpha }\phi _0\Vert _0\le \delta (i,\alpha )$.)

#### Lemma 6.3

Under the assumptions of Theorem 5.1, there exist $a,b>0$, such that for every $j\in \mathbb {N}$ and $t>0$,

$$\begin{aligned} {\mathrm {\Pi }}\bigl (f: \Vert f^{(j)}-f\Vert _{}>t+ a j^{1/2-\alpha /d}\bigr )\le e^{-bt^2j^{2\alpha /d}}. \end{aligned}$$

For $t^2=n\varepsilon _{n}^2/(4bj_n^{2\alpha /d})$ the bound in the preceding lemma becomes $e^{-4n\varepsilon _{n}^2}$. Hence (18) is satisfied for

$$\begin{aligned} \eta _n\gtrsim \sqrt{n} \varepsilon _{n} /j_n^{\alpha /d}+ j_n^{1/2-\alpha /d}. \end{aligned}$$

Here we choose $\varepsilon _{n}$ the minimal solution that satisfies the direct prior mass condition (17), given in (29). Next we solve for $\eta _n$ under the constraints, (15) and (16). The first of these constraints, $j_n\le n\varepsilon _{n}^2$, shows that the first term on the right side of the preceding display always dominates the second term. Therefore, we obtain the requirements $j_n\le n\varepsilon _{n}^2$ and

$$\begin{aligned} \eta _n{} & {} \ge \sqrt{n}\, n^{-(\beta \wedge (\alpha - d/2) + \gamma )/( 2\alpha + 2 \gamma )},\\ \eta _n{} & {} \ge n^{-(\beta \wedge (\alpha - d/2) + \gamma )/( 2\alpha + 2 \gamma )}j_n^{\gamma /d},\\ \eta _n{} & {} \ge j_n^{-\beta /d}. \end{aligned}$$

Depending on the relation between $\alpha$ and $\beta + d/2$, two situations arise:

1. $\alpha < \beta + d/2$. We choose $j_n \simeq n^{d/(2\alpha +2\gamma ) } = n\varepsilon _{n}^2$ and then see that the first two requirements in the preceding display both reduce to $\eta _n \ge n^{-(\alpha - d/2)/(2\alpha + 2\gamma )}$, while the third becomes $\eta _n\ge n^{-\beta /(2\alpha +2\gamma )}$ and becomes inactive.
2. $\alpha > \beta + d/2$. We choose $j_n \simeq n^{d/(2\alpha +2\gamma )}\le n\varepsilon _{n}^2$, and then see that all three requirements reduce to $\eta _n\ge n^{-\beta /(2\alpha +2\gamma )}$.

### Proof of Theorem 5.3

Theorem 5.3 is a corollary of Theorem 3.2, with the choices

$$\begin{aligned} \begin{array}{ll} \eta _n \simeq n^{-\beta /(2\beta +2\gamma + d)}, &{} \qquad \varepsilon _{n} \simeq n^{-(\beta + \gamma )/(2\beta + 2\gamma + d)},\\ j_n\simeq n\varepsilon _{n}^2 = n^{d/(2\beta + 2\gamma +d)},&{} \quad \eta _{n,\tau }\simeq \tau \, n^{(d/2-\alpha )/(2\beta +2\gamma +d)}. \end{array} \end{aligned}$$

Conditions (14), (15), and (16) are satisfied for these choices. Condition (24) is exactly the same as in the continuous observation scheme and is verified in Gugushvili et al. (2020). It remains to verify (17) and (23).

For (17), we use that $\Vert Af-Af_0\Vert _n\simeq \Vert \mathcal {I}_n(Af-Af_0)\Vert$ and next (30). The middle term on the right is $o(\varepsilon _{n})$. Therefore, it suffices to show, for some $\tau _{n}$,

$$\begin{aligned} \int _{\tau _{n}}^{2\tau _{n}} {\mathrm {\Pi }}_{\tau }\bigl ( \Vert \mathcal {I}_n Af - Af \Vert + \Vert Af - Af_0\Vert <\varepsilon _{n}\bigr )\,dQ(\tau )\ge e^{-n\varepsilon _{n}^2}. \end{aligned}$$

In Gugushvili et al. (2020) this inequality is shown to be true without the term $\Vert \mathcal {I}_n Af - Af \Vert$, for $\tau _{n}=n^{-(\alpha -d/2-\beta )/(2\beta +2\gamma +d)}$, and where we can replace $\varepsilon _{n}$ by $\varepsilon _{n}/2$. Thus it suffices to show that

$$\begin{aligned} \sup _{\tau _{n}<\tau <2\tau _{n}}{\mathrm {\Pi }}_{\tau }\bigl ( \Vert \mathcal {I}_n Af - Af \Vert >\varepsilon _{n}/2\bigr )=o(e^{-n\varepsilon _{n}^2}). \end{aligned}$$

Because ${\mathrm {\Pi }}_{\tau }$ is a scaling of ${\mathrm {\Pi }}={\mathrm {\Pi }}_1$, the left side is bounded above by ${\mathrm {\Pi }}\bigl ( \Vert \mathcal {I}_n Af - Af \Vert >\varepsilon _{n}/(2\tau _{n})\bigr )$. By Lemma 6.1 this is $o(e^{-n\varepsilon _{n}^2})$ if $(\varepsilon _{n}/\tau _{n})^2 n^{(2s+2\gamma )/d}\gg n\varepsilon _{n}^2$, where $s<\alpha -d/2$ can be chosen arbitrarily close to $\alpha -d/2$. (As noted in the preceding section, it can be chosen so that $s+\gamma >d/2$, since $\alpha +\gamma >d$, by assumption.) This leads to the inequality $\alpha +\gamma >(d/2)(2\beta +\gamma +d)/(2\beta +2\gamma )$, which is implied by the assumptions $\alpha +\gamma >d$ and $\beta +\gamma >d/2$.

For the proof of (23), we employ (31). As the continuous time equivalent that replaces $f^{(j,n)}$ by $f^{(j)}$, is shown to be true in Gugushvili et al. (2020), it suffices to show that, for every $\tau$,

$$\begin{aligned} {\mathrm {\Pi }}_{\tau }\Bigl (\frac{1}{\delta (j_n,\gamma )}\Vert \mathcal {I}_nAf-Af\Vert _{}>\eta _{n,\tau }\Bigr )\le e^{-4n\varepsilon _{n}^2}. \end{aligned}$$

By Lemma 6.1 this true if $(\eta _{n,\tau }/\tau )^2j_n^{-2\gamma /d}n^{(2s+2\gamma )/d}\gg n\varepsilon _{n}^2$. Since $(\eta _{n,\tau }/\tau )j_n^{-\gamma /d}\simeq \varepsilon _{n}/\tau _{n}$, this reduces to the same inequality as in the preceding paragraph.

## Appendix A Galerkin Projection

In this section we state for easy reference two results on the Galerkin method. Proofs may be found in the literature, or exactly in the present form in Gugushvili et al. (2020).

### Lemma 6.4

If $V_j$ is a finite-dimensional space as in Assumption 2.2 such that (6) and (7) hold, then, for $P_j: H_0\rightarrow V_j$ the orthogonal projection onto $V_j$, and $0\le s,t< S$,

A1 $$\begin{aligned} \Vert f - P_j f\Vert _{-t}{} & {} \lesssim \delta (j, t)\delta (j,s) \Vert f\Vert _s,\qquad f\in H_0,\end{aligned}$$

A2 $$\begin{aligned} \Vert g\Vert _s{} & {} \lesssim \frac{1}{\delta (j, s)\delta (j, t)} \Vert g\Vert _{-t},\qquad g\in V_j. \end{aligned}$$

### Lemma 6.5

If $V_j$ is a finite-dimensional space as in Assumption 2.2 such that (6) and (7) hold, and $A: H_0\rightarrow G$ is a bounded linear operator satisfying $\Vert Af\Vert _{} \simeq \Vert f\Vert _{-\gamma }$ for every $f\in H_0$, then the norms of the operators $R_j: G\rightarrow H_0$ and $R_j A: H_0\rightarrow H_0$ satisfy

A3 $$\begin{aligned} \Vert R_j\Vert _{}{} & {} \lesssim _A \frac{1}{\delta (j,\gamma )},\end{aligned}$$

A4 $$\begin{aligned} \Vert R_j A\Vert _{}{} & {} \lesssim _A 1. \end{aligned}$$

Furthermore, for $f\in H_s$ the Galerkin solution $f^{(j)}\in V_j$ to *Af* satisfies

A5 $$\begin{aligned} \Vert f^{(j)} - f\Vert _{} \lesssim _{A} \delta (j,s)\, \Vert f\Vert _s. \end{aligned}$$
